# Using mobile health to encourage physical activity in individuals with intellectual disability: a pilot mixed methods feasibility study

**DOI:** 10.3389/fresc.2023.1225641

**Published:** 2023-08-24

**Authors:** Henriette Michalsen, André Henriksen, Gunn Pettersen, Gunnar Hartvigsen, Silje Wangberg, Gyrd Thrane, Reidun Jahnsen, Audny Anke

**Affiliations:** ^1^ Department of Rehabilitation, University Hospital of North Norway, Tromsø, Norway; ^2^ Faculty of Health Sciences, Department of Clinical Medicine, UiT—The Artic University of Norway, Tromsø, Norway; ^3^ Faculty of Science and Technology, Department of Computer Science, UiT—The Artic University of Norway, Tromsø, Norway; ^4^ Faculty of Health Sciences, Department of Health and Care Sciences, UiT—The Arctic University of Norway, Tromsø, Norway; ^5^ Faculty of Health Sciences, Department of Health and Care Sciences, UiT—The Arctic University of Norway, Narvik, Norway; ^6^ Institute of Health and Society, Research Centre for Habilitation and Rehabilitation Models and Services (CHARM), Faculty of Medicine, University of Oslo, Oslo, Norway

**Keywords:** intellectual disability, physical activity, mobile health app, technology, mixed methods, activity trackers

## Abstract

**Background:**

Many individuals with intellectual disability (ID) have a sedentary lifestyle. Few interventions aimed at increasing their level of physical activity (PA) have shown lasting effects.

**Aim:**

To assess the feasibility and acceptability of a pilot intervention study using innovative mobile health (mHealth) support systems to encourage PA in individuals with ID.

**Methods:**

Nine individuals with ID and a low level of PA, aged 16–36 years, were included in the present convergent triangulation mixed method design. Two mHealth support systems (apps) were developed and tested. PA was measured with a Fitbit smartwatch, accelerometer, the International Physical Activity Questionnaire—Short Form (IPAQ-S), and Goal attainment scaling. Data were collected through online pre-, mid- (4 weeks), and post-intervention (12 weeks) questionnaires and activity trackers. Semi-structured qualitative interviews with participants and/or a family or staff member were held after the 12-week follow-up. Data were analyzed using conventional nonparametric statistics and thematic analyses.

**Results:**

The response rate and retention to the trial were 16% and 100%, respectively. Data quality was high, except for missing data from Fitbit activity trackers of approximately 30% from the 4- and 12-week follow-up stages. The feasibility challenges with activity trackers include rashes, size, non-acceptance, and loss of motivation. Participants and family members/staff reported interest in the study theme and were pleased with the data collection method. All but one participant achieved their PA goals. Most participants reported being satisfied with the apps as they were enjoyable or provided a reminder for performing physical and other activities. Social support for PA among family members also increased. However, app support from staff and family members was needed, and apps were not used regularly. Two of nine participants (22%) had increased their PA measured as steps per day with Fitbit at the 12-week follow-up.

**Conclusions:**

The acceptability and feasibility of using tailored mobile applications in natural settings to increase PA among adults with ID are promising. This study aligns with previous studies in showing the challenges to increasing PA, which requires the inclusion of family members, staff, and stakeholders. The intervention requires modifications before a randomized controlled trial can be conducted.

## 1. Introduction

Considerable evidence shows that physical activity (PA) yields numerous benefits for individuals with mild and moderate intellectual disability (ID) (1). Reported benefits include health advantages, such as increased cardiovascular and muscular capacity (2), while inconsistent results are found for improved social network and mental health (3). However, individuals with ID are less physically active than the general population (3, 4), and evidence for the intervention effects of improving PA levels is inconsistent (5, 6). Recent studies show that individuals with ID engage in more sedentary activities compared to the general population (3). A study comparing PA levels between individuals with and without ID found that adults without ID engaged in more light activities and had less sedentary time (7). Only 9% of adults with ID achieve the recommended levels of minimum 150 min of moderate-to-vigorous physical activity (MVPA) (4), compared to one out of five in the general population (8). Counting self-reported PA, about 63% of the general population reached recommended PA levels (9). Using steps per day as a measure, 7%–45% of the ID population reach a level of 100,000 steps per day (4). Developing methods to limit sedentary time and increase activity at any level can considerably improve health and reduce mortality among individuals with ID (10, 11).

The use of technologies to improve levels of PA has been explored to some extent. Lancioni et al. (12) published a scoping review of programs using stimulation-regulating technologies to promote PA in people with intellectual and multiple disabilities. Fifteen of the 42 studies included used video games (e.g., Wii gaming, virtual reality, Xbox, Light Curtain devices). None of the other 27 studies used mobile applications to promote PA in ambulatory adults with ID. Pérez-Cruzado and Cuesta-Vargas (13) published a pilot randomized controlled trial with four people (age undisclosed) with mild ID in the intervention group. The intervention was education, followed by reminders of PA through a mobile app with questionnaires as outcome measures. Martinez-Millana et al. (14) developed a motivating mobile app for indoor cycling and investigated user acceptance; however, no measures of PA were included.

In Norway, many individuals with ID have a smartphone or tablet that can be used for tailored PA interventions; however, this has not been tested in clinical studies. A previous study showed that individuals with ID are motivated to participate in PA and show an interest in technology (15). We have not found any previous studies promoting PA with the use of mobile apps and activity trackers to objectively measure levels of PA in adults with ID. Other studies have shown the measurable benefits of using mobile technologies for health-related behaviors and everyday life for individuals with ID (15–21). Few applications are available for promoting PA in individuals with ID, and the development of such technology in PA promotion is needed.

In the ID population, many studies have used objective PA measurement (22–24). Accelerometers are often the preferred measure, with both hip and wrist placements (22). Few studies have used commercialized activity trackers as an objective measure of PA in the ID population (25). Dario et al. (26) investigated the feasibility of using accelerometers together with the International Physical Activity Questionnaire—Short (IPAQ-S). Results showed that there were substantial agreements between reports on being active or inactive between the more acceptable and user friendly IPAQ-S and accelerometer data. However, IPAQ-S use has been found to both underestimate (4) and overreport levels of PA (27), compared to the accelerometer-measured PA levels.

According to the World Report on Disability, health promotion efforts targeting this population can improve lifestyle behaviors and these individuals have the right to be included in all PA programs (28). Specifically, a flexible approach is important when including individuals with complex cognitive challenges in health research (29). Testing procedures and interventions in pilot trials can improve the chances that a large-scale study will successfully achieve its objectives and perhaps lead to successful practical implementation (30). Additionally, using a mixed methods design can expand and strengthen the conclusions of a study (31). To increase the possibility of promoting PA in adults with ID, it is necessary to develop interventions with innovative applications.

This study aimed to investigate the feasibility and acceptability of a pilot intervention study using innovative applications developed to encourage PA in adults with ID. In this pilot study using a mixed methods approach, feasibility was investigated quantitatively and qualitatively through recruitment, trial retention, and completeness of data, and through the missing data analysis. Acceptability was explored qualitatively through satisfaction with the study procedures, activity measurement, and mobile applications.

## 2. Methods

### 2.1. Study design

**Figure 1:**
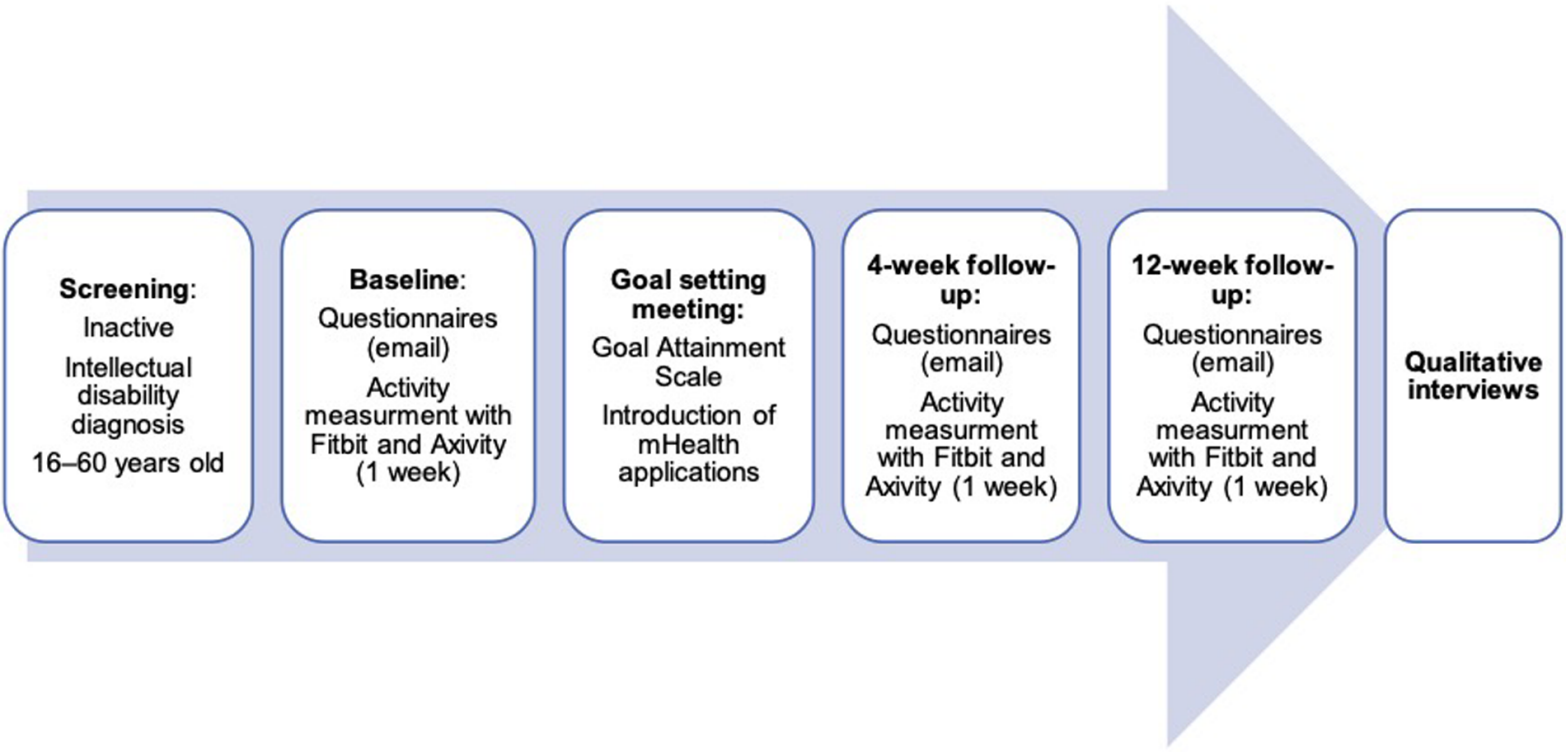
Pilot mixed methods study design.

A prospective pilot feasibility study with a concurrent triangulation mixed method approach (32) was carried out. Figure 1 provides an overview of the study procedures.

#### 2.1.1. Ethical considerations

The study was sought from and granted approval by the Regional Committees for Medical and Health Research Ethics in Norway (number 2016/1770) and by the data protection officer at the University Hospital of North Norway. The study included an intervention directed at a vulnerable group and proceeded cautiously. When possible, informed consent was obtained from the individuals with ID, if the person had the decision-making capability to consent. In addition, in the case the person with ID was unable to consent, a close relative provided the informed consent on behalf of the person with ID. The participants are informed that they may withdraw from the study at any time without consequences for the treatment.

### 2.2. Procedure and recruitment

Individuals had to possess the following characteristics to be eligible for the study: (1) ICD-10 (International Classification of Disease, 10th revision) diagnosis of ID (mild, moderate, severe, or profound); (2) low levels of PA (specified under); (3) aged 16–60 years, (4) no medical reason not to increase PA; (5) capable of walking with or without support; (6) capable of providing written informed consent if not obtained from a legal representative; and, (7) living in the municipality of Tromsø, Norway.

Individuals with ID were recruited over a 6-month period starting from May 2021. They were identified through their participation in the Norwegian Health in Intellectual Disability (NOHID) study (33), and through staff leaders at the municipal level, who identified potential participants. Research nurses from the clinical trial unit of the University Hospital of North Norway were responsible for data collection and storage. The second author (AH) was responsible for outlining the procedures for setting up and securing the registration of data from the activity measurements.

Invitation letters were sent to 74 participants from the NOHID study database, through a local day care center and high school. Letters were distributed to potential participants by post or handed to leaders of the day care center and high school, with no follow-up after they were sent. After receiving signed informed consent from the participants and/or a family member, the research nurses contacted a family member or staff member from the group home and completed the screening. The Physical Activity Readiness Questionnaire (34) was used to screen for medical contraindications to participation. The participants' carer or a staff member was asked the question, “How much of (the participants’) leisure time has (they) spent being physically active in the last year?” The four response categories were (1) participating in hard training or sports competitions regularly more than once a week, (2) jogging and other moderate sport or heavy gardening for at least four hours each week, (3) walking, cycling, or other forms of light exercise at least four hours a week, or (4) reading, watching TV or other sedentary activities. The question has been used in the surveys for PA in the general population (35) and the ones including individuals with ID (33, 36). If participants were reported doing mainly light PA (response category 3) or primarily sedentary activities (response category 4), they were included in the study.

For participants who passed the screening, baseline conversations were held over the telephone with a family or staff member immediately after the screening. Questionnaires were sent securely via e-mail, using the electronic system Research Electronic Data Capture (REDCap). This is a web-based system that is compliant with relevant regulations and security requirements. In case of missing questionnaire data, the family or staff member was contacted and given a reminder. Two activity trackers (Fitbit and Axivity), to be worn for 7 consecutive days, were handed to participants. According to the instructions, Fitbit was worn on the non-dominant hand and Axivity on the dominant hand.

After the baseline assessment, all participants were invited to a meeting with the main author (HM) to set goals for PA using the Goal Attainment Scaling (GAS) (37). During this meeting, participants were given a smartphone (iPhone) if they did not have one, with the developed applications (hereafter “apps”) installed. If they had their own phones, the researcher installed the apps included in the intervention on them. The researcher inserted all the usual PAs or leisure activities into one of the apps (an activity planner) through a web app and added the new activities defined in the GAS. The follow-up after four weeks included a phone call from the research nurses with questions about how they experienced participation, how the technology was working, and whether there were any problems with the activity trackers, and an e-mail containing the follow-up questionnaires. The same procedure was followed for the 12-week follow-up. The apps were available for use for 12 weeks after their introduction. All participants and their family or staff members were asked to participate in a qualitative interview after the 12-week assessment. The time and place for the interviews were agreed upon between the family or staff members and the first author (HM), who also conducted all interviews.

### 2.3. Application development

This pilot study was part of a project aimed at developing and testing innovative apps that promote PA in individuals with ID (14, 38, 39).

**Figure 2:**
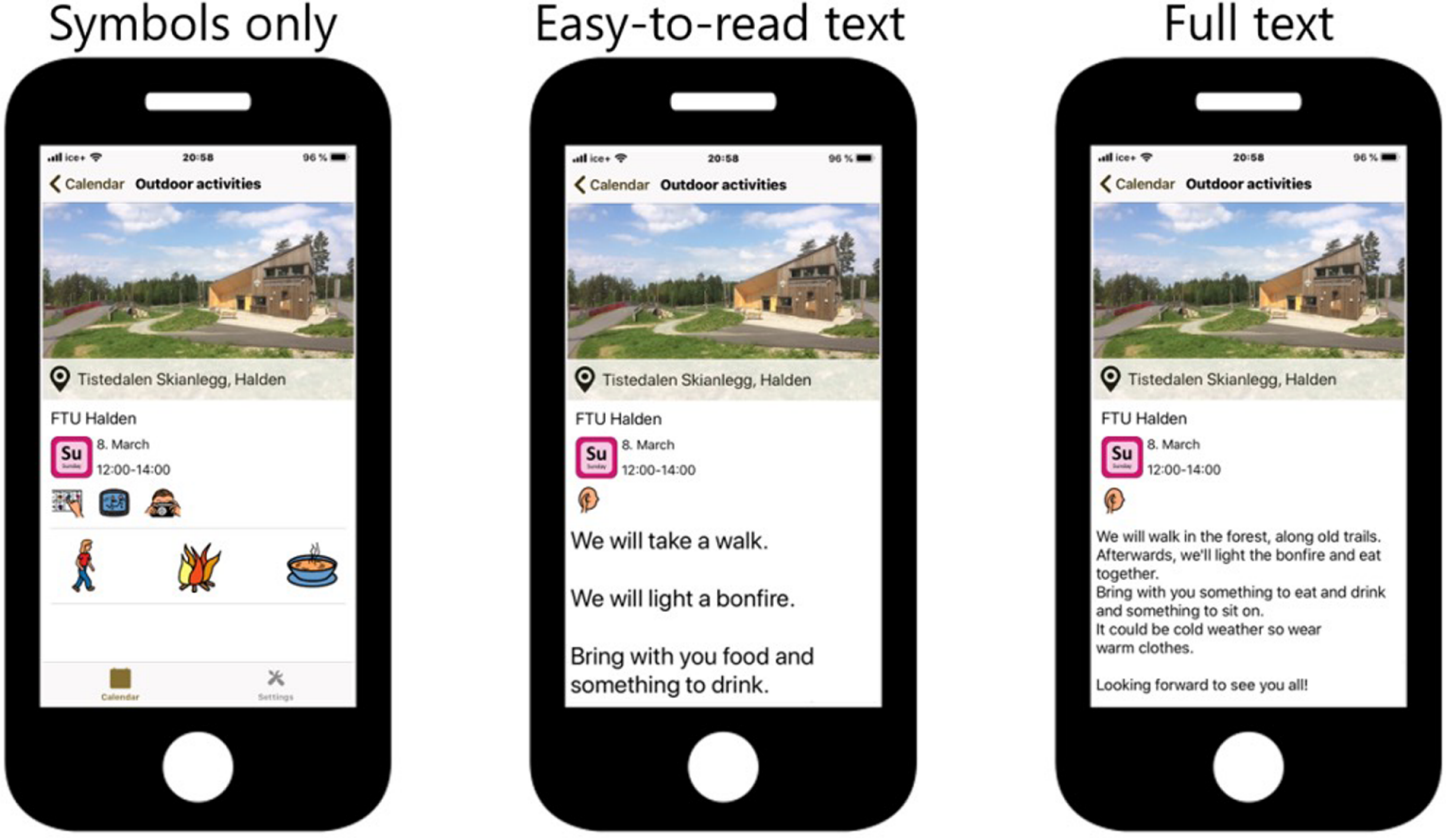
Interface options of the Active Leisure app: symbols only, easy-to-read text, or plain text. The app also has read-aloud capabilities.

The main app used in this pilot trial was named Active Leisure (Norwegian: *Aktiv Fritid*). It consists of an advanced adjusted activity planner based on a platform developed by the organization Smart Cognition AS (Smart Cognition AS, Norway), a non-profit business where profits are given as grants to projects contributing to better living conditions for people with disabilities. The developers were close family members of individuals with ID. In the development process, feedback was given from the user representatives in our reference group, as well as from the experts in the research group. The app offers individualized solutions for activities that are presented with pictures. Various interface options are available for tailoring (symbols only, easy-to-read text, plain text, or read-aloud), as shown in Figure 2. After completing the activity, a simple reward was provided (e.g., a smiling face or shareable picture). All the activities added to the activity planner were inserted through a web application. The activity planner was mostly used together by individuals with ID and a support person (family member or healthcare provider). Although this app is an off-the-shelf solution, Smart Cognition implemented (and included in the standard application) the following features specifically for our needs: possibility to register activities, simple rewards when registering activities as completed, possibility to add new, pre-defined activities in the mobile app.

An mHealth exercise app was also developed that could be added to the Active Leisure planner. This app is called Sorterius (40) and is an augmented reality game inspired by the popular game Pokémon Go. The idea for the app came from a previous qualitative study (15), and it has been discussed and presented in reference groups consisting of family members and staff of individuals with ID. At one of the reference group meetings, an individual with ID was present to test the prototype.

Sorterius was conceptualized and implemented as part of a Master's thesis project in computer science (41) during the spring of 2021. At the time, Covid restrictions prevented us from testing the game among people with ID. However, we conducted usability tests among eight people working with people with ID to improve the game before it was used in the present study. More details about the implementation of the game can be found in the thesis (41).

**Figure 3:**
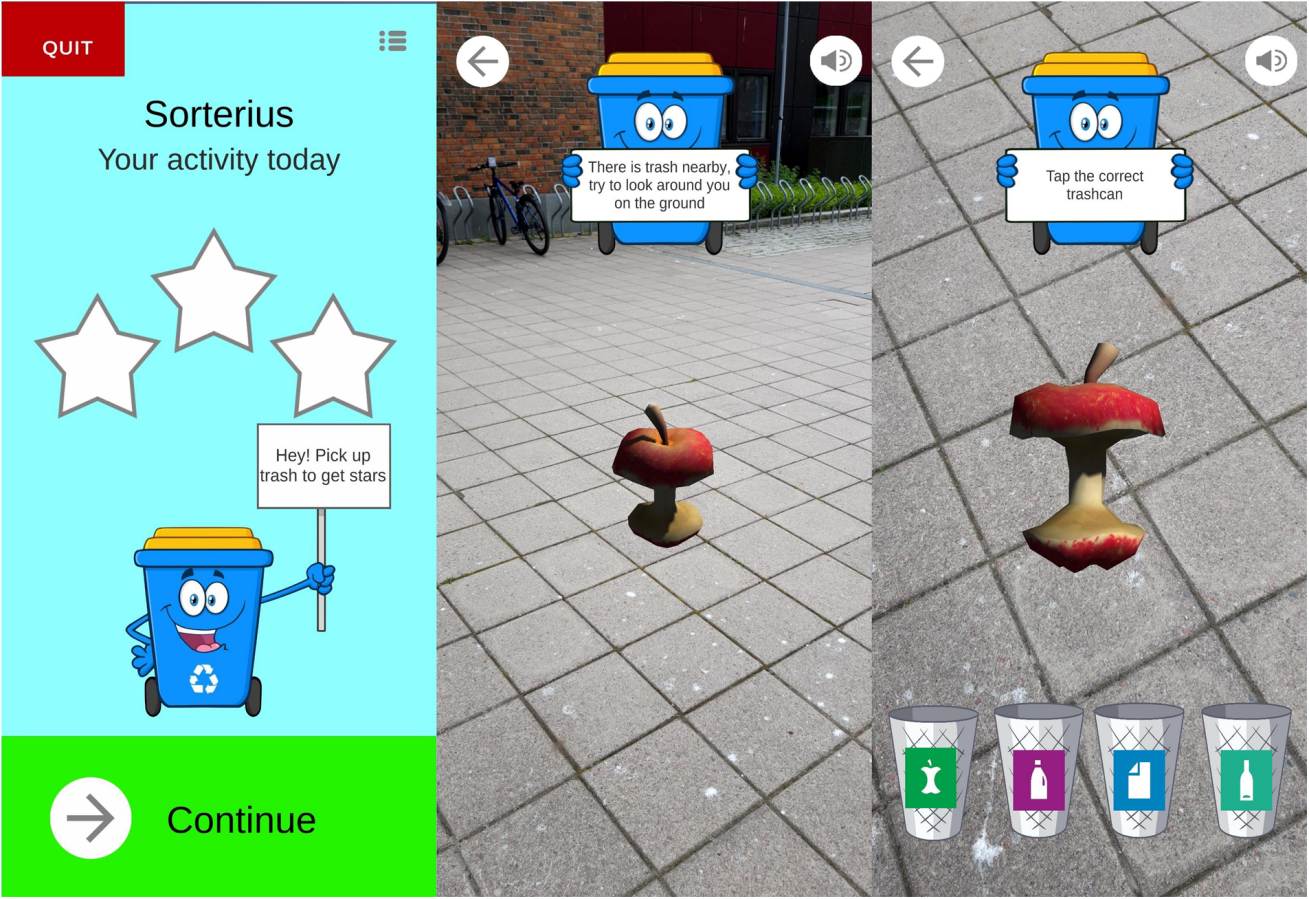
The augmented reality app “Sorterius” (42).

In this app, individuals walk in the real world while using a mobile phone. Through the camera of the phone, the individual observes virtual waste appear on the ground. The waste can then be picked up (i.e., clicked) by the player, whose task is to sort the waste into the correct waste bins, e.g., plastic waste goes into the plastic bin. There are three difficulty levels, and depending on the level chosen, the individual is presented with one (easy difficulty), two (medium difficulty), or four bins (hard difficulty). When a set number of items is collected, the individual receives a virtual reward (e.g., stars, and positive feedback). Adding goals for the steps per day, as well as a weekly goal is possible and could be tailored to each individual. A screenshot from the app is shown in Figure 3.

Sorterius is under continuous development. The version used in the current projects is freely available for Android (https://play.google.com/store/apps/details?id=no.uit.ifi.sorterius) and iPhone (https://apps.apple.com/us/app/sorterius/id1610130479).

### 2.4. Goal-setting meeting

During the goal-setting meeting, participants and their caregivers or staff members provided information about their current activities. All participants formulated two or three goals to increase their PA, together with their caregiver or staff member. The new goals were selected (43), formulated, and added to the Goal Attainment Scaling (44). Observable behaviors that reflected the degree of goal attainment were defined. Five different goal attainment levels, ranging from “no change,” “goal achievement” to “much better than expected outcome” (numbered −2 to +2, while 0 is goal achievement), were used. For example, one female participant went swimming once a month, which was defined as a score of −2 at baseline. Her new goal was to go swimming once a week (goal achievement, score of 0). By the 12-week follow-up, she had gone swimming once a week (the defined goal), and achieved a score of 0 on the GAS, indicating goal achievement. Another example was that one participant did not have any planned PAs during the weeks of summer (score −2) and set a goal to walk to and from the grocery store twice a week to buy bread. During the summer months, the participant went to the grocery store and back at least thrice a week, which indicated a “better than expected” outcome (score of 1).

The achievement of the goals set in the GAS was discussed during qualitative interviews.

### 2.5. Measures

An overview of the outcome measures used in the study is presented in Table 1 (45).

**Table 1:** Outcome measures used in the study.

Measurement tool	Measuring	Type of measure
Activity trackers		
Steps per day	PA, activity trackers	Primary outcome
Questionnaires International Physical Activity Questionnaire	Levels of PA	Secondary outcome
EuroQol-5D	Health-related quality of life	Secondary outcome
Aberrant Behavior Checklist–Community	Challenging behavior	Secondary outcome
Community Integration Questionnaire	Integration in community	
The Self-Efficacy/Social Support for activity for persons with intellectual disability scale	Self-efficacy and social support in PA	Secondary outcome
Goal attainment scaling (GAS)	Goal achievement	Method, secondary outcome

PA, Physical activity.

#### 2.5.1. Activity measurement with activity trackers

All participants were asked to wear a Fitbit Versa (Fitbit LLC, CA, US) smartwatch on the non-dominant wrist and an accelerometer, Axivity X3X (Axivity Ltd, Newcastle, UK), on the dominant wrist. Participants who only agreed to use one of the activity trackers chose the Fitbit device, as PA output from this device will be used to assess main study outcomes in a later definite trial. The choice of having two activity trackers was based on the idea of doing a comparison study of the devices in the ID population later in the research project. Except for days of valid measurement, data from the Axivity device are not presented in the current study.

The use of Fitbit for objective PA measure has not yet been validated in the ID population but has been used in an intervention study for individuals with ID and autism (25). The accuracy of using Fitbit has been tested in a rehabilitation population (46). We provided several choices regarding the device color, size, and color and material of the band. Devices were distributed on the same day as the baseline assessments. Participants had to wear the Fitbit device for at least three consecutive days, with a minimum of 500 steps per day for the measurement of steps per day to be valid (47).

In this study, steps per day from the Fitbit device were the main outcome. Data from the Axivity device will be analyzed and used later.

### 2.6. Questionnaires

The included questionnaires in the pilot study were chosen as possible individual, interpersonal or environmental correlates to participation in physical activity or sedentary behavior (3), such as aberrant behavior, communication, health related quality of life, living situation, self-efficacy/social support for PA, and integration in the community. PA was measured with a questionnaire due to known problems with missing data on accelerometers (5).

Information regarding age, sex, and living conditions was collected. Living situations were classified as living independently, living with family, or living in a group home with care (48). Information regarding the degree of ID was obtained from participants' medical records. The degree of ID was categorized as mild (IQ: 50–69), moderate (IQ: 35–49), severe (IQ: 20–34), or profound (IQ: <20) (49). The Communication Function Classification System (CFCS) (50) was used to register communication levels.

The International Physical Activity Questionnaire—Short Form (IPAQ-S) was used to measure proxy-reported PA levels (51, 52). The IPAQ-S is a 7-item questionnaire that assesses PA in the past seven days at four intensity levels: (1) vigorous-intensity activity, such as aerobics; (2) moderate-intensity activity, such as leisure cycling; (3) walking; and, (4) sitting. It was scored as a continuous measure by calculating the volume of activity based on its energy requirements, defined in metabolic equivalents (METs), to yield a score in total MET minutes per week (53). Per the IPAQ-S scoring instructions, reaching between 1,500 and 3,000 MET minutes per week is defined as having high PA levels, between 600 and 1,500 is moderate, and under 600 MET minutes is defined as insufficiently active or inactive. This scale has been validated in the general population (54), and substantial agreement between instruments was found in a feasibility trial in the ID population (26). The same study found excellent agreement between IPAQ-S scores from participants with ID and their proxies.

To measure health-related quality of life, the generic EuroQol-5D-5l (EQ-5D-5l) was used (55). The scale is divided into five areas/items: mobility, self-care, usual activities, pain/discomfort, and anxiety/depression. Each item is scored from 1 to 5, where 1 indicates no problem in performing a task and 5 indicates an inability to perform a task. The overall index score was calculated based on the normal values of a population of Nordic participants without ID (56). No index scores have been found for the ID population. The index score is reported between zero and one, and scores closer to one indicate a higher health-related quality of life. The feasibility of using this scale in research that includes individuals with ID has been explored, with a high proportion experiencing difficulties in answering (57). The EQ-5D can be completed via a proxy respondent who know the person well (58), and the 5l version is validated for proxies of people with dementia (59).

To assess challenging behavior, the Aberrant Behavior Checklist-Community (ABC-C) was used (60). The checklist consisted of 58 items divided into five subscales: irritability, social withdrawal, stereotypy, hyperactivity, and inappropriate speech. It is a proxy measure that requires the knowledge of the person with ID. Each item is scored on a scale of 0–3 (with 3 indicating the most severe). The questionnaire was validated for use in a Norwegian population with neurodevelopmental disabilities (61).

The Community Integration Questionnaire (CIQ) was used to obtain information on how connected participants were in their communities (62). The CIQ consists of 15 items related to home and social integration, and productive activities. The scores were 0, 1, or 2 depending on the level of integration, with a maximum total score of 12, indicating a high level of community integration. This scale is developed for persons with aquired brain injury and can be completed by self-report or by a close caregiver (62). Promising psychometric properties for people with other disabilities have been found (63).

To assess self-efficacy and social support in a PA setting, the Self-Efficacy/Social Support for Activity for Persons with Intellectual Disability Scale (SE/SS-AID) was used (64). It is a questionnaire consisting of four subscales: the first (6 items) measures self-efficacy for overcoming barriers to leisure PA, and the last three measure social support for leisure activities from family members (7 items), care staff (6 items), and friends of individuals with IDs (5 items). The scale has been validated for self-reporting and use by proxy respondents in the ID population (64) and translated into Norwegian using standard guidelines (65).

The GAS was reported as normalized T-scores. A mean score of 50 with a standard deviation of 10 corresponded to the achievement of the goal (score of 0) (44). The scale has been validated as having good responsiveness and sensitivity to change (66) and has been used in studies including individuals with ID (31).

### 2.7. Feasibility

The feasibility measures included recruitment, adherence to the study, adherence to the use of apps and activity measures, and data quality, which were assessed as a percentage of missing data. Recruitment was assessed by (1) response rate, the proportion of participants who provided written consent for the number of invitations sent out; and, (2) inclusion rate, the proportion of individuals included from the number of consenting ones.

Completeness of data was defined as a percentage of missing questionnaire data, percentage of non-participation in goal-setting meetings, and qualitative interviews. In addition, reasons for missing data were explored in the qualitative material.

### 2.8. Acceptability

Acceptability of the trial methods and intervention was assessed via qualitative interviews. In line with other studies (67), acceptability was defined as satisfaction with the study as a whole (procedures, contact, and information), satisfaction with the measurement of PA by activity trackers, and satisfaction with the use of the apps.

The qualitative interviews were held after the 12-week assessment. They were semi-structured using an interview guide categorized into two sections. Section one focused on feasibility and acceptability of procedures, the use of activity measurement, how the mHealth support was used, and participant, caregiver or staff experiences in all aspects of the study. Section two focused on technology and motivation for physical activity, and will be analyzed in a later publication.

The interviews were audiotaped, and then transcribed verbatim and anonymized. The interviews lasted from 20 min to 2.5 h. The interviews were held at the participants' preferred places: home (*n* = 6), the day center they attended (*n* = 1), or the hospital (*n* = 2).

### 2.9. Data analysis

In this study, quantitative and qualitative data were gathered and analyzed separately. In the final interpretation of the results, data from both methods were brought together with the qualitative data supplementing the quantitative data.

Appropriate quantitative statistical analyses were performed using the SPSS 28 software (IBM Corp.) according to the type and distribution of data. The descriptive statistics were presented as medians with interquartile ranges, means with standard deviations, 95% confidence intervals, and frequencies of categorical data. The distribution properties of the variables were also examined. Following the CONSORT 2010 extension, estimates of the effects of participant outcome measures (from baseline to follow-up) were explored using nonparametric statistics (related-sample Wilcoxon Signed Rank Test) (68). A tendency toward change with a significance level of 10% was reported. The minimal clinically important individual difference in steps per day was defined as a 10% change from baseline to follow-up (47).

The transcribed interviews were analyzed using thematic analysis (69). The interview transcripts were read several times by the first author to identify emerging themes. Data on the use of activity measurement, use of technology (in general and in using the apps), and experiences of participation in the research project were selected and further analyzed. The text from the transcripts was transformed into specific codes. The codes were compared based on differences and similarities, and condensed into meaningful categories and subcategories (69, 70). The preliminary analysis was read and commented on by the authors, AA and GP. Subsequently, following discussions among the authors, the main themes were identified by grouping similar subthemes and linking them to the results of the quantitative analysis of feasibility.

Mixed analyses were also conducted after quantitative and qualitative data were gathered and analyzed separately (32). In the final interpretation of the results, data from both methods are brought together and supplement each other. In this study, quantitative data were supplemented by information in the qualitative material. For instance, in the event of missing data, interviews have shed light on why there are more missing data at one point of measurement than at others. The quantitative data analysis was performed independently of the qualitative analysis (32).

## 3. Results

### 3.1. Feasibility

This pilot study aimed to include ten participants (39). In total, 12 individuals of the 74 invited provided signed informed consent, resulting in a response rate of 16%. The remaining 12 individuals were screened for participation. Two individuals did not meet the inclusion criteria of a low level of PA and one dropped out before the baseline assessments. This meant that nine individuals with ID participated at baseline, which gives an inclusion rate of 75% of those who consented.

All nine participants (100%) who were included in the study took part in goal-setting meetings and qualitative interviews, resulting in a 100% retention rate. From the questionnaires, all (100%) were filled out at baseline, eight (88%) at the 4-week follow-up, and nine (100%) at the 12-week follow-up. Data quality, assessed as a percentage of missing data in each received questionnaire, was <1%.

Days of valid measurements (minimum of three days of measurement) for the Fitbit device showed that all nine (100%) participants had valid measurements from baseline, five (66%) at the 4-week follow-up, and seven (77%) at the 12-week follow-up. For the Axivity, days of valid measurement were seven (77%) at baseline, six (66%) at the 4-week follow-up, and five (55%) at the 12-week follow-up.

Missing data analysis from the qualitative data showed that at the 4-week follow-up one participant lost motivation and threw both measurement devices in the trash. For the second participant, who wore the Fitbit device longer than one week and charged the device with a private charger, data was not possible to retrieve when the device was sent back. The same participant lost motivation at the 12-week follow-up and did not wear any of the devices. The third participant got a rash from the metal and rubber band on the Fitbit device and wore only the Axivity at the 4- and 12-week follow-ups. The fourth missing at 4-weeks follow-up had small wrists and both devices were too large. It was still possible to retrieve some of the data at the 12-week follow-up, but there were uncertainties about the quality of the activity data.

Data retrieved from the Fitbit measurement did not display wear-time or how much time the participant spent sleeping. There were also more missing data when looking at intensity of the PA for the 4-week and 12-week follow-up than for the step count. It was thereby difficult to analyze activity data using intensity categories (sedentary time, light, moderate, and vigorous) from the Fitbit measurements. From the Axivity device, wear-time and time spent in different levels of activity were available, but not analyzed. Data from the Fitbit device were defined as the main outcome in the current pilot trial.

In the qualitative interviews, participants with ID attended six out of nine interviews. Only two of the six participants were active throughout the interviews. In three interviews, only a family member or staff member participated.

### 3.2. Participant characteristics

The participants' personal characteristics are listed in Table 2. The mean age of the participants was 27 years (SD = 7.25), and seven participants were female. Four participants attended day center activities, and four worked either regularly or with support. Most participants had a moderate level of ID. All participants walked without aid or support. Two participants could communicate effectively with both known and unknown communication partners, but had slower progress in their speech. Two individuals had articulation difficulties and could communicate effectively only with their known communication partners. The remaining participants did not have any communication difficulties.

**Table 2:** Participant characteristics.

	Participants
	*N* = 9
Age, years	
Median (range)	28 (19–30)
Gender, *n* (%)	
Female	7 (77)
Male	2 (22)
Level of ID, *n* (%)	
Mild	1 (11)
Moderate	8 (88)
Severe/Profound	0
Occupation, *n* (%)	
Regular paid work	1 (11)
Work with support	3 (33)^a^
Day center activity	4 (44)
Attending school	2 (22)
Living situation, *n* (%)	
Lives independently	1 (11)
Lives with family	2 (22)
Apartment in group home with care	6 (67)
CFCS, *n* (%)	
Level 1	5 (56)
Level 2	2 (22)
Level 3	2 (22)
Level 4–5	0

ID, intellectual disability, CFCS, Communication Function Classification System.

^a^ Some individuals attended both schools and worked with support.

### 3.3. Estimation of possible effects

#### 3.3.1. Physical activity measured as steps per day

The participants' PA levels from the Fitbit device (steps) are presented in Table 3. The median (IQR) steps for the participants were 5,080 (3,269–7,251) at baseline (*n* = 9), 7,734 (4,770–9,176) at the 4-week follow-up (*n* = 5), and 5,057 (1,968–7,240) at the 12-week follow-up (*n* = 7). A numeric tendency toward an increase in mean steps per day from baseline to the 4-week follow-up was based on five participants, three with a clinically important increase in steps per day. Of the seven participants with measurements at baseline and the 12-week follow-up, two showed a clinically important increase in PA. Estimations of possible changes revealed no overall differences in steps per day between the time points. In terms of individual steps per day, one participant had more than 10,000 mean steps at baseline, six had approximately 5,000 mean steps or more, and one had less than 2,000 mean steps per day.

**Table 3:** Mean steps per day for each participant at baseline, 4 weeks, and 12 weeks, with minimal clinical important changes.

ID	Mean baseline (*N* = 9)	Mean 4-week (*N* = 5)	Mean 12-week (*N* = 6)	Change baseline—4-week (10% or more)^a^	Change baseline—12-week (10% or more)^b^
1	5,080	8,395	11,083	Increase	Increase
2	4,966	4,015	3,869	Decrease	Decrease
3	6,652	5,527	6,259	Decrease	No change
4	4,507	7,734	4,380	Increase	No change
5	7,851	0	0	–	–
6	5,118	9,956	7,248	Increase	Increase
7	12,228	0	0	–	–
8	1,900	0	1,968	–	No change
9	4,255	0	1,728	–	Decrease
Total mean	5,516	7,126	5,315		
95% confidence interval	3,564–6,387	4,198–10,053	3,278–10,125		
Total median	5,080	7,734	5,057		
IQR	3,269–7,251	4,770–9,176	1,968–7,240		

^a^ Change baseline—4-week = mean steps 4-week—mean steps baseline; change is minimum 10%.

^b^ Change baseline—12-week = mean steps 12-week—mean steps baseline; change is minimum 10%.

#### 3.3.2. GAS

A positive change occurred in goal attainment between the goal-setting meeting and end of the study, with a 10% significance level (*p* = 0.085) (Table 4). Goal achievement equals a T-score of 50, and the median score for evaluation of the GAS score at the end of the study is 45.4 (IQR: 37.7–51.4) for the whole group. Four participants set three goals, and the rest set two goals to increase PA. Only one participant had no goal achievement at the end of the study, seven had goal achievement (0) or better than expected (+1) for one or more goals, and one had much better than expected (+2) for one of the two goals.

**Table 4:** Results from questionnaires.

Measure	Baseline sum *N* = 9, median (IQR)	4-week sum *N* = 8 median (IQR)	12-week sum *N* = 9, median (IQR)
IPAQ-short	947 (286–1,377)	825.6 (421–1,179.3)	660 (186–1,391.3)
CIQ	15 (11.8–17.3)	16 (13.3–17.4)	15 (13.4–18.6)
SE/SS-AID			
Self-efficacy PA	6,5 (6–8.8) *N* = 8	8.5 (3–11.5)	8 (4–11)
Social support family*	**10** **(7.5–12)**	**12** **(10.5–14)**	**12** **(0–6)** *N* = 8
Social support staff	8 (5.5–11.5)	7 (4.3–11.8)	10 (5.5–11.5)
Social support friends	6 (2.5–8)	4 (1.3–9.5)	4 (0.5–7.5)
ABC-C			
Irritability	1 (0–5)	0.5 (0–3.3)	1 (0–3.5)
Social withdrawal	5 (2.5–10)	4.5 (0.5–8.3)	4 (0.5–7)
Stereotypic behavior	0 (0–4)	0 (0–2.8)	0 (0–2)
Hyperactivity	4 (1.5–4.5)	1.5 (0.3–2)	1 (0–6)
Inappropriate speech	1 (0–2)	1 (0–2.3)	1 (0–3.5)
EQ5D	0.78 (0.68–1)	0.78 (0.73–0.92)	0.78 (0.66–0.92)
GAS*	**37.6** **(36.3–37.6)**		**45.4** (**37.7–51.4)**

IPAQ, International Physical Activity Questionnaire; CIQ, Community Integration Questionnaire; SE/SS-AID, Self-Efficacy/Social Support Activities for Persons with Intellectual Disability; ABC-C, Aberrant Behavior Checklist-Community.

B1, Self-efficacy in physical activities; B2, Social support from family members; B3, Social support from staff; B4, Social support from friends.

* The bold values are the significant results (*p* < 0.10).

#### 3.3.3. Estimation of possible changes in questionnaires

There was an increase in social support from family members for PA (subscale B2 in the SS-AID) between baseline and the 4-week follow-up (*p* = 0.017), and from baseline to the 12-week follow-up (*p* = 0.074). There was otherwise no statistically significant difference between measures at baseline and follow-up, or between measures from the 4-week follow-up to the 12-week follow-up (Table 4). Median MET-minutes from the IPAQ-S were reported as decreasing from baseline to the 4- and the 12-week follow-up, but no statistically significant difference (using non-parametric tests). Table 5 displays the results on only the PA-related measurements.

**Table 5:** Results from the different measures of physical activity behavior.

ID	Fitbit Steps/day	IPAQ-s METs	GAS score
1			
Baseline	5,080	4,050	36.3
4-week	8,395	496	52.7
12-weeks	11,083	891	
2			
Baseline	4,966	735	37.58
4-week	4,015	1,072	43.8
12-weeks	3,869	0	
3			
Baseline	6,652	148.5	37.58
4-week	5,527	396	43.8
12-weeks	6,259	505.5	
4			
Baseline	4,507	1,017	37.58
4-week	7,734	–	25.15
12-weeks	4,380	148.5	
5			
Baseline	7,851	946,5	36.3
4-week	–	1,306.5	43.43
12-weeks	–	1,297.5	
6			
Baseline	5,118	1,737	37.58
4-week	9,956	661.1	56.21
12-weeks	7,248	372	
7			
Baseline	12,228	990	36.3
4-week	–	990	45.43
12-weeks	–	3,075	
8			
Baseline	1,900	424	37.58
4-week	–	1,215	50
12-weeks	1,968	660	
9			
Baseline	4,255	0	36.3
4-week	–	198	31.74
12-weeks	1,728	0	

METs, Metabolic Equivalent minutes per week; GAS, Goal Attainment Scaling.

Missing data is reported with a line (–).

A decrease in hyperactivity symptoms from the ABC-C was observed from baseline to both the 4-week and 12-week follow-ups (displayed in Table 4), without the changes being statistically or clinically significant.

### 3.4. Acceptability

Aspects of acceptability were analyzed into two main themes: “positive experiences” and “areas of improvement.” In the presentation of the qualitative results, themes and codes from the analysis have been structured so that they corresponded to the study's aim and definition of acceptability, rather than according to the individual themes and codes.

#### 3.4.1. Satisfaction with the applications

Almost all participants achieved one or two goals based on the GAS measurement. All participants had their goals added to the activity planner (Active Leisure) app and had the option to register their accomplished activities.

Everyday structure and fun: The participants, family members, and staff reported that the activity planner (Active Leisure) was easy to use and that it was interesting to receive rewards when registering activities. The activity planner provided a structure for the leisure activities of the participants and reminded them to focus more on physical activities in their daily routines. When using the app, participants had more predictability of the new activities that they were going to perform. Seeing future activities through pictures, symbols, and text in the app also offered predictability of what the week would consist of regarding physical and leisure activities.

Reminder for inclusion: The app also helped the staff remember to include the participants in other activities, such as social and cultural activities. One family member administered the activity planner herself and included all the participants' daily activities, besides PA, in the Active Leisure app.

*“He found it interesting opening the app and seeing all the things he was supposed to do and what time he should do it. We had not only added physical activities but all other things to do during the day and added things that he is very interested in…sometimes it’s hard to make him do things and then it is much better to add them to a plan and then he thinks like okay this is something I must do…”*—*mother*

Lack of information: Many staff members working in shifts, as well as summer substitutes in the group homes, did not receive information about the project, and therefore did not use the apps. Family members helped the participants with the apps at the beginning of the project, but they found it difficult to add or change activities to the plan when required.

Supplement for daily life: The waste sorting app was used as a supplement when walking from one location to another. This gave the walking activity a goal and greater meaning. They also enjoyed the familiarity of the waste that appeared on the screen and were excited when they received a reward after sorting the waste.

*“…it (the app) talks a lot. But I found a Zalo bottle (Norwegian dish soap) and a conditioner on the ground!”—participant with ID*

They found the game to be less interesting when the time between waste appearances was too long, and could lose the motivation to continue playing.

Need of support: The results indicated that both apps were primarily used by family members or staff together with the participants. Only one participant used both apps independently (one with high cognitive function).

#### 3.4.2. Satisfaction with activity trackers

As shown in the quantitative data, all participants used the activity trackers during baseline assessments. The participants liked that the activity trackers had different colors and were used as accessories; some were keen on showing the activity trackers to others, in the hope of receiving positive feedback.

Previous experiences: At the 4-week follow-up, there were more missing data from the activity trackers. Two participants had previous experiences using activity trackers and were disappointed when the research-adjusted Fitbit device did not provide the feedback they had previously received. It was also reported that the loss of motivation could be because the information assigned to the participants beforehand was incomprehensible, and they did not know why they were supposed to wear them.

*“…as we have discussed among staff, we believed that he simply threw both measurement devices in the bin and then took the trash out for recycling. His room is so tidy and organized that there is no way he could have misplaced the devices…”—Staff member*

Adjustments: In several cases, the activity trackers caused skin irritation and adjustments had to be made, such as switching to fabric wristbands or placing sweatbands underneath. However, the skin irritation did not always seem to reduce motivation, as some of those who had skin irritation at baseline continued to wear the device after adjustments were made (at the 4- and 12-week follow-ups).

#### 3.4.3. Satisfaction with study procedures

All participants participated in a goal-setting meeting and qualitative interview, and only one participant had missing questionnaire data at the 4-week follow-up. This indicates a high retention rate and adherence to the study. The qualitative findings have provided supplementary information.

Flexible approach: Participants perceived the study as interesting and important, and most individuals with ID, family members, or staff were pleased with the method of data collection. The method of collecting data was reported to be both flexible and varied, helping family members or staff to maintain motivation and feel satisfied with participation.

*“This is such an important theme, and I really wanted her to take part in this—even though it was an added everyday effort for us to participate.”*—*father*

Important study focus: All family members and staff were pleased with the focus of the study (PA) and wanted to contribute to the research on this subject. The participants themselves said that they thought it was good to participate in the project and were pleased with the researcher visiting them at home. Family members and staff found distance data sampling (emails and phone calls) an easy way of answering questions and liked that they were given reminders to fill out the questionnaires.

*“When you come to us, it is very easy. Also, getting the devices delivered has been nice… ”*—*mother*

Lack of information: Some staff requested more information about the study from the group home or daycare center before the study was initiated. They believed that more participants would have wanted to participate if the project had been prioritized by closer leaders at group homes rather than more distant ones at the municipality level.

*“…if the information about the study and participation came from the group home leaders, this would create a better acceptance for the time and resources spent doing activities with Lisa.”—Staff member*

They also mentioned that the time of data collection (4-week follow-up during summer) created problems for participation or the use of the apps. This was due to less staff availability and fewer resources to perform activities with the participants. Some family members and staff also requested more information to the participants that they themselves could understand.

## 4. Discussion

The purpose of this pilot trial of a 12-week pilot goal-directed PA intervention with mobile application support in adults with ID, was to investigate the feasibility and acceptability of the intervention. The main findings show excellent adherence to the study and data quality for questionnaires, although objective PA measurements were missing for one-third of the participants at follow-up. Eight of nine participants achieved goal attainment for PA, and two individuals exhibited an increase in PA by the end of the study. Furthermore, qualitative results showed positive experiences in using the applications. Participants and family members/staff reported an interest in the study theme and were pleased with the method of data collection, with an estimated statistically significant increase in social support from family members for PAs. However, the recruitment rate was relatively low, which aligns with other studies (26).

Some clinical intervention studies that aim to increase PA show similarly high data quality for questionnaires and retention (43), but many intervention studies on PA report missing data (23, 24). From the activity measurement using Fitbit wrist-worn wearables, missing data were found at the 4-week and 12-week follow-ups, which was also found in other studies, including objective measures of PA using accelerometers or pedometers (70, 71). The quality of the activity data can be questioned, as wear time for the Fitbit device was not retrieved. Qualitative data confirmed that there were issues related to wearing the measurement devices (Fitbit and Axivity) related to skin irritation or loss of motivation, which has been found in other studies (26).

Leung et al.'s (22) systematic review from 2017 showed that 22 studies included accelerometers to objectively measure PA in individuals with ID. In most studies, the accelerometers were placed on the hip. None of the included studies used wrist-worn accelerometers or commercial activity trackers. Wrist-worn accelerometers are not as accurate in providing estimates of energy expenditure but often have better acceptability and higher wear-time (72). Qualitative data from the current pilot study showed satisfaction with using activity trackers at baseline assessments; however, issues regarding skin irritation, size, non-acceptance, and loss of motivation were apparent at follow-up. The absence of missing data from the baseline assessments could imply that wrist-worn accelerometers and/or commercial activity trackers can be useful for high activity data quality in future trials. Increased monitoring during follow-up could potentially be beneficial in avoiding missing data from activity measurements.

Tendencies towards discrepancies between levels of PA objectively measured as steps per day with Fitbit and subjectively as proxy-reported METs from IPAQ-S are seen. This aligns with Moss & Czyz's (73) study, but contradicts the findings of another study (26) that found substantial agreement between the objective and subjective measures in determining active or inactive behaviors. However, none of the measures showed significant changes in total PA from baseline to follow-up. An apparently high median score of METs at baseline is mainly due to a high score from one participant, who may have misunderstood the questionnaire. The IPAQ-S is, as reported earlier (26), more acceptable than an activity tracker, and thereby more suitable for individuals with severe and profound ID. Also, step count at baseline indicate a higher activity level than the inclusion criteria of less than four hours walking per week. It could be that the one-week baseline assessment with activity trackers motivated for a higher activity level than at the timepoint of the screening.

Nearly all participants in this study achieved one or two goals for the PA set using the GAS. Using goal attainment as part of a PA intervention in adults with ID has not been conducted in other studies, to the best of our knowledge. In a study of 92 children with disabilities by Willis et al. (43), GAS was used as one of the outcome measures, and only 32% of participants showed goal attainment for a PA goal at the 12-week follow-up. Combining goal-setting with technology by adding goals to a digital activity planner is promising. The results of the qualitative data showed a positive attitude toward using this specially developed and adjusted digital activity planner that creates structure and predictability. It reminded staff and family members about inclusion and planning for PAs, and may be used together with individuals with ID to increase engagement. Activity measurement data showed a clinically significant increase in PA for only two participants; however, the high rate of goal attainment could have positively influenced the PA of the remaining participants. All types of increases in PA are regarded as positive health outcomes (10).

Few studies have a technological intervention focusing on the structure and predictability of PA. In several studies, predictability is an important facilitator for PA (74–76). In the scoping review by Lancioni et al. (12) of stimulation-regulating technology to increase PA, one group of studies (*n* = 15) used computer video games (77, 78). The majority of the included studies (*n* = 27) used sensors or other stimulation-regulating technology linked to computer systems or mobile technology to increase PA in the form of increased balance, stretching, and arm, leg, and head responses. None of the studies in the last group used wrist-worn activity trackers or accelerometers to measure the steps per day as the outcome (12). Three of the included studies with eight, nine, and six participants used smartphones to increase PA with positive results. However, in contrast to the present study, the participants had severe or profound ID with severe motor or vision impairments and were not ambulatory (19, 20, 79, 80). In the present pilot study, the results indicated satisfaction with the apps used in the present pilot study; the apps were easy to use and sparked interest in the participants with ID. A previous promising study of reminders for PA through a mobile app (13) was performed with four individuals with mild ID and evaluated using the IPAQ. This differs from the current study, in which eight of nine participants had moderate ID, and PA was monitored objectively in addition to using the IPAQ.

Furthermore, this study aimed to develop and evaluate mobile apps for PA (39). One reason that development was necessary was that none of the available apps for PA had inclusive designs intended for adults with ID. An inclusive design includes a simple interface, text alternatives, sufficient contrast, navigational help, and robust systems (81). Communication was also a focus of the apps developed and adjusted for in this pilot trial. None of the studies we read involved increasing PA in adults with ID had the option of using a digital planner for PA, nor did they have an inclusive design as a focus in technological interventions. Using mobile technology for activity planning improves availability and accessibility, as many family members, support persons, and staff own a smartphone (82). Planning also involves engagement from staff and family members, which has been another important predictor of the facilitation of PA for individuals with ID (74, 75). One finding of the qualitative interviews was that the apps were not used independently or over time. Finding ways for these mobile apps to improve the engagement of staff or family members will be crucial for future development.

The present results showed an increase in social support for PA from family members after four and twelve weeks. This indicates that either the study procedures or intervention positively impacted family member engagement in PA. Two individuals who had family members as their support person during the study showed a clinically significant increase in PA. This further emphasizes the importance of creating engagement and interest in PA among persons supporting individuals with ID (74). The estimated effects showed no change in social support for PA among staff members.

### 4.1. Modifications before a future mHealth PA intervention

Some staff members requested more planning, information, and involvement from leaders and stakeholders of the study to improve its procedures. Future studies should consider ways on improving the support and engagement of staff when developing interventions. Others have been successful with PA interventions, in which either staff or caregivers have been included to conduct or instruct PA interventions (23, 71, 83). The use of family members or staff as mentors in PA interventions may be important to ensure long-term changes in PA behavior (5). Although the research project had a reference group of user representatives and experts, a more formalised inclusive research design could increase the recruitment rate. Goal-directed intervention in combination with an inclusive activity planner is a promising approach that should be investigated in randomized controlled studies. In future trials, a multicenter approach should be used to ensure recruitment from a larger population of adults with ID. In addition, objective measures of how much mHealth apps are used and the wear-time for activity trackers should be included in a future trial.

### 4.2. Limitations and strengths

This pilot mixed methods feasibility trial has several limitations. The small sample size evidently reduces the generalizability of the findings; however, nine out of ten planned participants were included in this pilot (39). Strengths of the pilot trial include originality as the first mobile-based intervention for PA in ambulatory adults with ID, objectively measuring PA, and the use and evaluation with a mixed methods design, as well as the use of commercially-available activity trackers. Missing data from activity trackers at approximately one-third of the follow-up points was another limitation, a problem also found in other PA intervention studies (24). Furthermore, the activity measurement may not reflect the actual activity of the participants. Typically, a day of 10-hour wear-time is considered a valid day for measurement (84). In this study, at least 500 steps per day (23), were required to be considered a valid measurement, which could overshadow missing step counts. Another possibility is that the achieved activity goals were not measured during the three days of valid measurements (e.g., swimming once a week), but other studies have defined a three-day period with at least 6 h' measurement a day as valid (23). Wear-time for the Fitbit measurement has not been obtained, which is another important limitation that needs to be addressed in future trials.

In this study, no objective measures or back-end recordings of the time spent on the two different apps during the intervention existed. Apps were reported to be used more frequently at the beginning of the study than regularly throughout the study. It is not uncommon for people to lose interest in PA apps after the novelty of the technology has worn off (84), but this has not been extensively investigated for individuals with ID.

Most participants included in the study had moderate ID. Future trials should include more individuals with severe or profound ID to investigate how the use of mobile applications can be adjusted to increase PA. The low recruitment rate may indicate a possible selection bias of participants who are particularly interested in the research topic. Another limitation was that most of the participants were female, which does not provide a balanced view of the gender differences in the general ID population (6). In future trials, a more equal distribution (or more males) in the included participants should be ensured.

## 5. Conclusion

This is the first study (to the best of our knowledge) to examine the feasibility and acceptability of a pilot PA intervention study using specially developed mobile apps coupled with wrist-worn activity trackers in adults with intellectual disability. The acceptability and feasibility of using goal attainment combined with tailored mobile applications to increase PA are promising. A full study should include participants from a larger area and aim for more engagement from staff and stakeholders.

## Data Availability

The datasets presented in this article are not readily available to protect the anonymity of the participants. Requests to access the datasets should be directed to the authors.
